# Population-based prevalence, incidence, and disease burden of autoimmune hepatitis in South Korea

**DOI:** 10.1371/journal.pone.0182391

**Published:** 2017-08-03

**Authors:** Bo Hyun Kim, Hwa Young Choi, Moran Ki, Kyung-Ah Kim, Eun Sun Jang, Sook-Hyang Jeong

**Affiliations:** 1 Center for Liver Cancer, National Cancer Center, Goyang, Republic of Korea; 2 Department of Cancer Control and Policy, Graduate School of Cancer Science and Policy National Cancer Center, Goyang, Republic of Korea; 3 Department of Internal Medicine Inje University Ilsan Paik Hospital, Goyang, Republic of Korea; 4 Department of Internal Medicine, Seoul National University Bundang Hospital, College of Medicine, Seoul National University, Korea; Kaohsiung Medical University Chung Ho Memorial Hospital, TAIWAN

## Abstract

**Background and aim:**

Little is known about population-based epidemiology and disease burden of autoimmune hepatitis (AIH). The aim of this study was to investigate the prevalence, incidence, comorbidity and direct medical cost of AIH in South Korea.

**Methods:**

The data was from the nationwide, population-based National Health Insurance Service claims database and the Rare Intractable Disease registration program. Age and gender-specific prevalence rates were calculated, and data on comorbidity, diagnostic tests, prescribed drugs, and medical costs were retrieved for patients registered under the disease code K75.4 (AIH) from 2009 to 2013.

**Results:**

A total of 4,085 patients with AIH were identified between 2009 and 2013 with a female-to-male ratio of 6.4. The age-adjusted prevalence rate was 4.82/100,000 persons and gender adjusted prevalence rates were 8.35 in females and 1.30 in males. The age-adjusted calculated incidence rate was 1.07/100,000 persons (gender-adjusted 1.83 in females and 0.31 in males). Ascites, variceal bleeding, and hepatocellular carcinoma were found in 1.4%, 1.3%, and 2.2% of the patients, respectively. Forty-six patients (1.1%) underwent liver transplantation during the study period. Case-fatality was 2.18%. Corticosteroid and azathioprine were prescribed in 44.1% and 38.0% of prevalent patients with AIH in 2013, respectively. The nationwide total direct medical cost was less than 4.0 million USD, and the average cost for each patient was 1,174 USD in 2013.

**Conclusion:**

This is the first report on the nationwide epidemiology of AIH in Korea, and it showed a lower prevalence than that of Western countries with considerable disease burden.

## Introduction

Autoimmune hepatitis (AIH) is a rare, chronic liver disease characterized by the presence of auto-antibodies, hypergammaglobulinemia, and interface hepatitis on histological examination. AIH, unless treated properly, can progress to cirrhosis or hepatic failure. Nonetheless, the epidemiology, treatment pattern, and disease burden of AIH are difficult to determine because of diverse clinical presentations and low prevalence.

The disease prevalence and incidence of AIH differ by race and ethnicity. Prevalence estimates range widely from 4 to 42.9 cases per 100,000 persons [1–11], and reported annual incidences range from 0.67 to 2.23 cases per 100,000 persons.[1, 4, 6, 8–14] The variable results may be attributed to differences in genetics, environmental factors, and study population or design, and these heterogeneous factors make it challenging to understand the global epidemiology and disease burden of AIH.

A comprehensive study on the nationwide epidemiology and direct medical costs of AIH may be able to overcome selection biases related to patients and organizations when based on a population-based administrative database. This study used a claims data base from the Korean National Health Insurance (NHI) system, which is a mandatory nationwide insurance system operated by the government. This data set includes almost all of the population’s inpatient and outpatient healthcare utilization data (96.6% in 2010) and contains all medical and prescription drug-claim records. In addition, NHI has an established registry program for rare intractable diseases (RID), which includes AIH or primary biliary cirrhosis (PBC), for copayment reduction since 2009. To be registered to this RID system, physicians must confirm the specific diagnosis by checking the listed diagnostic criteria and then register their patients. The aim of this study was to investigate the nationwide, population-based epidemiology, clinical features (such as complications or comorbidities), and disease burden of AIH in South Korea using these two big databases.

## Patients and methods

### Data source

The present study used the nationwide NHI claims database and RID database, which contain inpatient and outpatient healthcare utilization information such as patients’ demographics, date of admission and discharge, date of visit, diagnostic procedures such as laboratory tests or imaging studies, surgical procedures, prescription history, principal diagnoses or comorbidities based on the International Classification of Disease, 10^th^ Revision (ICD-10), and RID registry information. Patients with RIDs become eligible for copayment reduction after their diagnoses are confirmed by a physician on the basis of the RID-defined diagnostic criteria provided by the NHI. Diagnoses are reviewed by the corresponding healthcare institution before being submitted to the NHI. All personal identities were encrypted and all data was analyzed anonymously. The cases of death were investigated from the data of Statistics Korea. This study was approved by the Institutional Review Board of the National Cancer Center (NCC2014-0182).

### Study population

Patients with the disease code of AIH (K75.4) between January 2009 and December 2013 were retrieved from the claims data of NHI and were matched with the RID registry program. In the RID registry program, AIH was identified through a clinical diagnosis based on the results of auto-antibodies, immunoglobulin G, or histological examination and differential diagnosis from other liver diseases such as viral hepatitis. A prevalent case was defined as a case with AIH as the primary or secondary diagnosis of the year along with records of visits, hospitalizations, or surgeries in the claims database of NHI. An incident case was defined as a newly registered one without any claims data for AIH during the washout period to exclude prevalent cases. A 2-year washout period (2009–2010) was used in this study; therefore, new cases from 2011 were those that had no claims data for AIH in 2009 and 2010.

### Statistical analyses

This study calculated the crude and adjusted prevalence of AIH in Korea during 2009 to 2013 using the number of AIH cases in the corresponding year divided by the population and by age- and gender-specific populations of the standard population. The standard population for the age- and gender adjusted prevalence rates was the registered population in 2010 by the Ministry of Government Administration and Home Affairs of the Republic of Korea.[15] The incidence rate was calculated by newly registered cases divided by the registered population. Cases of death from Statistics Korea were used for the case-fatality rate. All statistical analyses were performed using SAS 9.3 (SAS Institute, Cary, NC, USA), and a p-value <0.05 was considered to indicate a statistically significant difference.

## Results

### Prevalence and incidence of AIH in South Korea

From 2009 to 2013, a total of 4,085 AIH cases were registered after excluding duplicate cases. Among them, 3,493 were females (85.5%) and 592 were males (14.5%) with a female-to-male ratio of 6.4. The median case age was 56 years (56 for females and 55 for males), and the mean age was 54.9 years (55.1 for women and 53.2 for men).

**Fig 1:**
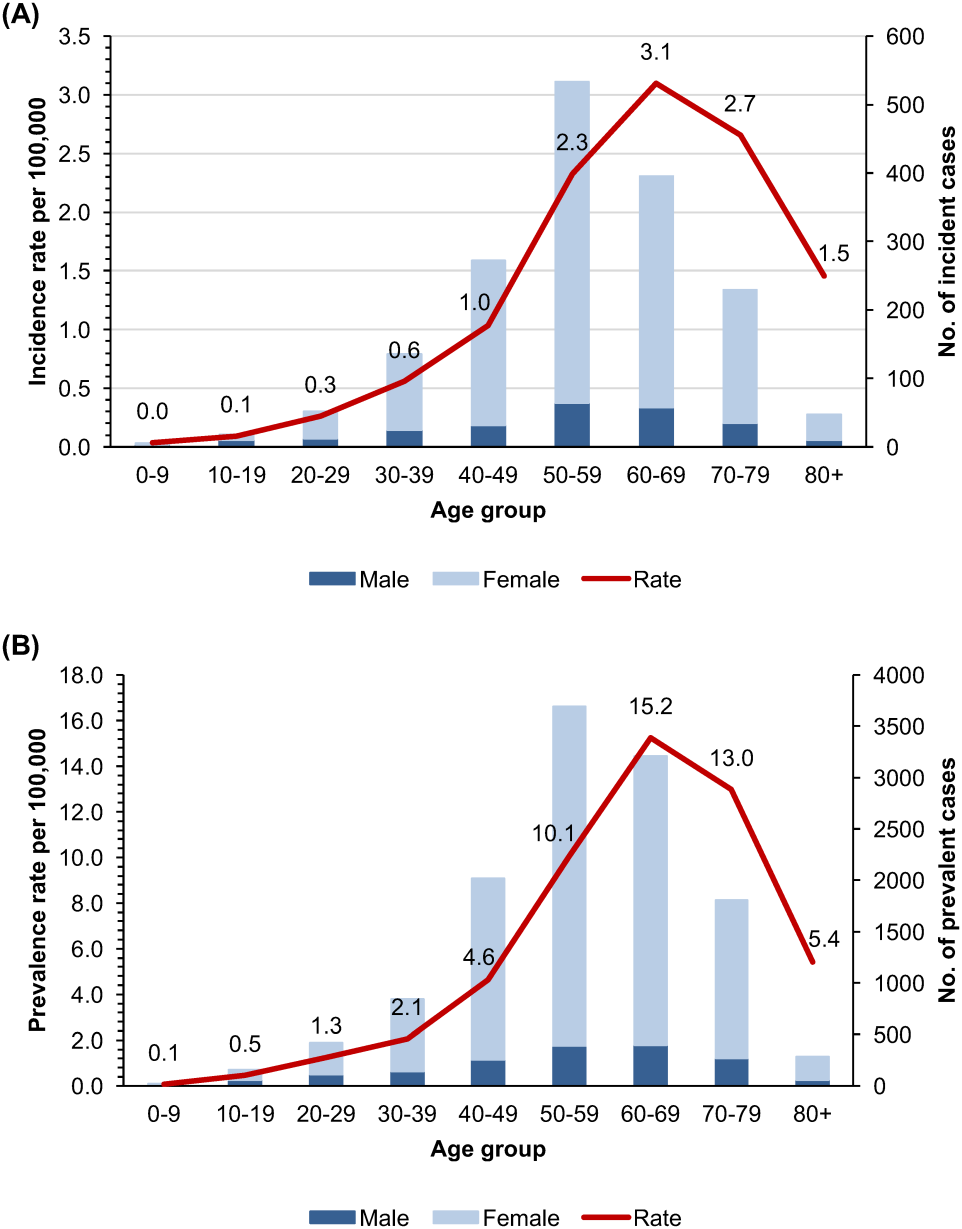
(A) Average annual gender-adjusted incidence rate per hundred thousand population and incident cases (2009–2013) of AIH by age in South Korea. (B) Average annual gender-adjusted prevalence rate per hundred thousand population and prevalent cases (2009–2013) of AIH by age in South Korea.

The average age-adjusted prevalence rate of AIH was 4.82/100,000 persons (gender-adjusted: 8.35 in females and 1.30 in males) during 2009–2013 (Table 1). The peak prevalence was in ages 60–69 (15.24/100,000, Fig 1) and was ages 60–69 for females (25.53/100,000) and 70–79 for males (4.62/100,000).

**Table 1:** Crude and adjusted prevalence and incidence rates per 100,000 of AIH in South Korea.

	Standard population*	Total number of cases	2009	2010	2011	2012	2013	Average, annual, adjusted rate
**Prevalence**								
Gender								
Male	25,035,384	1686	1.12	1.13	1.28	1.47	1.51	1.30†
Female	24,973,069	10,761	6.69	7.26	8.42	9.36	10.02	8.35†
Age (years)								
0–9	4757524	18	0.12	0.04	0.09	0.09	0.04	0.08‡
10–19	6826875	154	0.51	0.45	0.43	0.45	0.48	0.47‡
20–29	6,826,755	422	1.15	1.20	1.41	1.28	1.24	1.25‡
30–39	8,278,025	842	1.80	1.74	2.23	2.30	2.25	2.06‡
40–49	8,701,782	2,021	3.84	4.26	4.67	5.09	5.27	4.63‡
50–59	6,945,354	3,689	8.69	8.68	10.00	11.26	11.65	10.06‡
60–69	4,137,957	3,210	11.87	13.22	15.03	17.06	19.04	15.24‡
70–79	2,595,847	1,810	8.63	10.67	12.89	15.54	17.14	12.97‡
80+	938,334	281	3.31	4.05	5.41	6.43	7.91	5.42‡
Total	50,008,453	12,447	3.90	4.19	4.84	5.41	5.76	4.82§
**Incidence**								
Gender								
Male	25,035,384	249			0.32	0.36	0.27	0.31†
Female	24,973,069	1,438			1.91	1.87	1.70	1.83†
Age (years)								
0–9	4757524	5			0.04	0.06	0.00	0.04‡
10–19	6826875	18			0.09	0.11	0.08	0.09‡
20–29	6,826,755	52			0.42	0.15	0.22	0.26‡
30–39	8,278,025	135			0.61	0.58	0.48	0.56‡
40–49	8,701,782	272			1.14	0.99	0.99	1.04‡
50–59	6,945,354	533			2.30	2.72	1.97	2.33‡
60–69	4,137,957	396			2.82	3.42	3.06	3.10‡
70–79	2,595,847	229			3.04	2.37	2.56	2.66‡
80+	938,334	47			1.50	1.21	1.65	1.46‡
Total	50,008,453	1,687			1.11	1.12	0.98	1.07§

*Standard population, inhabitants in 2010

^†^Adjusted for age

^‡^Adjusted for gender

^§^Adjusted for age and gender

To determine the incidence accurately, the washout period was set for 2 years, and the incidence rate was calculated from 2011 to 2013. The overall age-adjusted AIH incidence rate during 2011–2013 was 1.07/100,000/year (gender-adjusted: 1.83 in females and 0.31 in males). Age-specific incidence rate was highest in ages 60–69 (3.10/100,000/year). By gender, it was the highest in ages 60–69 for women (5.07/100,000/year) and in ages over 80 (1.60/100,000/year) for men.

### Complications and case fatality of AIH in South Korea

ICD-10 code identified ascites was present in 1.4% of patients with AIH between 2009 and 2013, while diuretics (spironolactone) was prescribed in 5.8% of those with AIH (Table 2). ICD-10 code identified variceal bleeding was reported in 1.3% of patients with AIH. Endoscopic intervention for variceal bleeding was performed in 1.9% of patients, vasoactive medications such as terlipressin, somatostatin, or octreotide were given in 2.1%, and propranolol in 5.6% of patients during the 5 year study period. ICD-10 code identified hepatic encephalopathy was recorded in 2.2% of patients, and lactulose was prescribed in 12.3%. Hepatocellular carcinoma was diagnosed in 31 patients (0.8%). The median age of the 31 patients was 65 (range, 60–69), and 77.4% were females. Among them, 3 and 9 underwent surgical resection and local ablation, respectively. Liver transplantation was performed in 46 patients, and their median age was 53.5 (range, 43–61) and 89.1% were females.

**Table 2:** Proportion of AIH patients with complications or comorbidities from 2009 to 2013.

Complications or comorbidities	Male (n = 592, 14.5%)			Female (n = 3493, 85.5%)		
	0–29 65(11.0)	30–59 288(48.6)	60+ 239(40.4)	0–29 163(4.7)	30–59 1973(56.5)	60+ 1355(38.8)
**Complications**						
Ascites*	0(0.0)	2(0.7)	8(3.3)	1(0.6)	19(1.0)	26(1.9)
Prescription of spironolactone	2(3.1)	19(6.6)	9(3.8)	5(3.1)	114(5.8)	89(6.6)
Prescription of furosemide	2(3.1)	26(9.0)	45(18.8)	20(12.3)	184(9.3)	234(17.3)
Variceal bleeding*	1(1.5)	3(1.0)	1(0.4)	2(1.2)	25(1.3)	21(1.5)
Endoscopic intervention	0(0.0)	4(1.4)	1(0.4)	4(2.5)	33(1.7)	36(2.7)
TIPS†	0(0.0)	1(0.3)	0(0.0)	0(0.0)	0(0.0)	1(0.1)
Prescription of vasoactive agents	1(1.5)	8(2.8)	1(0.4)	4(2.5)	35(1.8)	38(2.8)
Prescription of propranolol	2(3.1)	12(4.2)	14(5.9)	10(6.1)	92(4.7)	97(7.2)
Hepatic encephalopathy*	0(0.0)	4(1.4)	8(3.3)	5(3.1)	37(1.9)	34(2.5)
Prescription of lactulose	4(6.2)	25(8.7)	30(12.6)	14(8.6)	219(11.1)	211(15.6)
Liver cirrhosis*	9(13.8)	69(24.0)	73(3.1)	31(19.0)	545(27.6)	596(44.0)
Hepatocellular carcinoma*	0(0.0)	1(0.3)	6(2.5)	0(0.0)	10(0.5)	14(1)
Liver resection	0(0.0)	2(0.7)	0(0.0)	0(0.0)	0(0.0)	1(0.1)
Local ablation	0(0.0)	0(0.0)	1(0.4)	0(0.0)	3(0.2)	5(0.4)
Transarterial chemoembolization	0(0.0)	1(0.3)	2(0.8)	1(0.6)	6(0.3)	8(0.6)
Liver transplantation*	1(1.5)	2(0.7)	2(0.8)	5(3.1)	24(1.2)	12(0.9)
**Comorbidities**						
Thyroid disease						
Hypothyroidism	2(3.1)	4(1.4)	2(0.8)	3(1.8)	68(3.4)	33(2.4)
Hyperthyroidism	0(0.0)	3(1)	0(0.0)	3(1.8)	22(1.1)	9(0.7)
Thyroiditis	0(0.0)	3(1)	0(0.0)	1(0.6)	22(1.1)	10(0.7)
Prescription of thyroid hormone	0(0.0)	13(4.5)	11(4.6)	2(1.2)	69(3.5)	53(3.9)
Prescription of anti-thyroid drugs	0(0.0)	1(0.3)	0(0.0)	3(1.8)	8(0.4)	3(0.2)
Autoimmune disease						
Primary biliary cirrhosis	1(1.5)	20(6.9)	13(5.4)	4(2.5)	173(8.8)	91(6.7)
Systemic lupus erythematosus	3(4.6)	2(0.7)	0(0.0)	6(3.7)	46(2.3)	9(0.7)
Systemic sclerosis	0(0.0)	0(0.0)	2(0.8)	0(0.0)	5(0.3)	1(0.1)
Rheumatoid arthritis	0(0.0)	1(0.3)	0(0.0)	0(0.0)	4(0.2)	6(0.4)
Dyslipidemia	1(1.5)	25(8.7)	38(15.9)	14(8.6)	165(8.4)	228(16.8)
Prescription of statin	3(4.6)	40(13.9)	26(10.9)	9(5.5)	288(14.6)	226(16.7)
Osteoporosis	0(0.0)	0(0.0)	0(0.0)	1(0.6)	4(0.2)	4(0.3)
Prescription of bisphosphonate	0(0.0)	0(0.0)	13(5.4)	0(0.0)	38(1.9)	100(7.4)

*Complications or comorbidities were identified with ICD-10 codes verified by physicians.

^†^TIPS denotes transjugular intrahepatic portosystemic shunt surgery.

N = 4085

During the 5 years of study, 271 of 4,085 patients died. The overall case-fatality ratio was 6.63% (6.38% for females and 8.11% for males). The average annual case fatality ratio was 2.18% (2.07% for females and 2.85% for males) as shown in Table 3.

**Table 3:** Case-fatality ratio (CFR, %)* for patients with AIH by gender and year in South Korea.

	2009 (n = 1899)	2010 (n = 2095)	2011 (n = 2487)	2012 (n = 2853)	2013 (n = 3113)	Five year CFR† (n = 4085)
Number (%)						
Female	21 (1.29)	40 (2.21)	53 (2.45)	54 (2.19)	55 (2.04)	223 (2.07)
Male	5 (1.82)	6 (2.13)	9 (2.74)	15 (3.85)	13 (3.16)	48 (2.85)
Total	26 (1.37)	46 (2.20)	62 (2.49)	69 (2.42)	68 (2.18)	271 (2.18)

* The case-fatality ratio was calculated by dividing the number of death cases in the corresponding year by the treated AIH cases.

^†^During 5 years (2009–2013), 271 of 4085 patients died, resulting in an overall case-fatality ratio of 2.18%

### Diagnosis and treatment for AIH in Korea

Of the 507 patients newly diagnosed with AIH in 2013, liver biopsy was performed in 54.2% of them. Tests for anti-nuclear antibody, anti-smooth muscle antibody, anti-mitochondrial antibody, and anti-liver kidney microsome type 1 antibody were done in 93.9%, 81.7%, 88.4% and 67.5% of patients, respectively. Among 3,113 prevalent AIH cases in year 2013, corticosteroid and azathioprine were prescribed in 44.1% and 38.0%, respectively.

### Comorbidities in patients with AIH

The most frequently encountered comorbid disease identified by ICD-10 codes and prescribed drugs was dyslipidemia (11.5%), which was followed by thyroid diseases (Table 2). Prevalence of comorbid PBC was found to be 7.4% in patients with AIH, suggesting an overlap syndrome. Systemic lupus erythematosus was also identified in 1.6% of AIH patients. Osteoporosis estimated by the prescription of bisphosphonate was present in 3.7% of patients.

Out of 302 patients diagnosed with AIH and PBC, 88.7% were females, and the median age was 54 (Table 4). Ascites and variceal bleeding identified by the ICD-10 disease code were more frequently found in patients with AIH-PBC compared to those diagnosed with AIH only. Liver transplantation was also performed more often in patients with AIH-PBC. However, reported frequencies of other comorbidities or HCC were comparable (Table 4).

**Table 4:** Comparison between patients with only AIH and AIH-PBC.

	Only AIH (n = 3783, 92.6%)		AIH & PBC (n = 302, 7.4%)	
	N	%	N	%
**Gender**				
Male	558	14.8	34	11.3
Female	3225	85.2	268	88.7
**Age (years)**				
0–9	12	0.3	0	0.0
10–19	60	1.6	0	0.0
20–29	150	4.0	6	2.0
30–39	304	8.0	24	7.9
40–49	643	17.0	75	24.8
50–59	1123	29.7	94	31.1
60–69	921	24.3	74	24.5
70–79	490	13.0	24	7.9
80+	80	2.1	5	1.7
Average	54.9		54.2	
Median (Q1, Q3)‡	56 (47, 65)		54 (46, 62)	
**Fatality in 2009–2013**				
Gender				
Male	45	8.1	3	8.8
Female	197	6.1	26	9.7
Age (years)				
Average	64.5		59.9	
Median (Q1, Q3)‡	67 (56, 75)		61 (55, 67)	
**Complications**				
Ascites*	65	1.7	12	4.0
Prescription of spironolactone	266	7.0	45	14.9
Prescription of furosemide	571	15.1	62	20.5
Variceal bleeding*	59	1.6	19	6.3
Endoscopic intervention	75	2.0	21	7.0
TIPS†	2	0.1	0	0.0
Prescription of vasoactive agents	100	2.6	15	5.0
Prescription of propranolol	225	5.9	32	10.6
Hepatic encephalopathy*	94	2.5	6	2.0
Prescription of lactulose	552	14.6	50	16.6
Liver cirrhosis*	1268	33.5	101	33.4
Hepatocellular carcinoma*	45	1.2	4	1.3
Liver resection	5	0.1	0	0.0
Local ablation	11	0.3	0	0.0
Transarterial chemoembolization	29	0.8	1	0.3
Liver transplantation*	50	1.3	7	2.3
**Comorbidities**				
Thyroid disease				
Hypothyroidism	140	3.7	8	2.6
Hyperthyroidism	44	1.2	8	2.6
Thyroiditis	48	1.3	5	1.7
Prescription of thyroid hormone	149	3.9	19	6.3
Prescription of anti-thyroid drugs	15	0.4	1	0.3
Autoimmune disease				
Systemic lupus erythematosus	85	2.2	9	3.0
Systemic sclerosis	12	0.3	5	1.7
Rheumatoid arthritis	17	0.4	1	0.3
Dyslipidemia	471	12.5	54	17.9
Prescription of statin	593	15.7	90	29.8
Osteoporosis	12	0.3	2	0.7
Prescription of bisphosphonate	166	4.4	13	4.3

*Complications or comorbidities were identified with ICD-10 codes verified by physicians.

^†^TIPS denotes transjugular intrahepatic portosystemic shunt surgery.

^‡^Q denotes quartile.

### Economic burden of AIH in Korea

The total national direct medical cost for AIH increased from 1,872,872 USD in 2009 to 3,654,099 USD in 2013 (Table 5). The annual direct medical cost per capita was 1,174 USD in 2013. Direct medical cost per capita was higher in men compared to that in women. All national medical costs for health services not covered by the NHI were excluded.

**Table 5:** Annual direct medical cost* of patients with AIH in 2013.

Age	Male			Female		
	N	Total direct medical cost (USD) †	Direct medical cost per capita (USD)	N	Total direct medical cost (USD)	Direct medical cost per capita (USD)
<20	14	15,859	1,133	18	35,897	1,994
20–29	24	17,180	716	57	61,167	1,073
30–39	27	41,899	1,552	152	154,718	1,994
40–49	60	44,017	734	405	394,693	975
50–59	93	230,780	2,482	825	884,611	1,072
60–69	99	169,394	1,711	734	854,979	1,165
70–79	79	99,628	1,261	435	520,100	1,196
80+	16	7,110	444	75	122,066	1,628
Total	412	625,868	1,519	2701	3,028,231	1,121

*Direct medical cost includes only cost reimbursed by the National Health Insurance.

^†^1 USD = 1150 KRW

## Discussion

The present study is the first nationwide epidemiology report on AIH in South Korea. The average age- adjusted prevalence of AIH between 2009 and 2013 was 4.8 per 100,000 persons, and the gender-adjusted prevalence rates were 8.4/100,000 for females and 1.3/100,000 for males. The average age-adjusted incidence of AIH was 1.1/100,000/year, and the gender- adjusted prevalence rates were 1.8/100,000/year for females and 0.3/100,000/year for males.

Compared to other populations, the incidence and prevalence of AIH in South Korea found in this study was quite low. The incidence and prevalence rates of AIH vary widely across different populations (Table 6).[1–14, 16] The prevalence was highest in an Alaskan native population (42.9/100,000), modest in European and New Zealand populations (mainly Caucasians, 10.7–24.5/100,000), and low in populations from Singapore and Brunei (4–5.6/100,000).[1–6, 9, 10] The annual incidence rate was low in Taiwan and Israel (0.52–0.67/100,000) and relatively high in Europe (0.85-3/100,000).[1, 4, 8–10, 12–14] Recently, a Japanese study reported a high prevalence and incidence of AIH (23.4/100,000 and 2.23/100,000/year, respectively).[11]

**Table 6:** Prevalence and incidence of AIH.

	Year	Region	Cases (no)	Prevalence period	Prevalence/100,000	Incidence period	Annual incidence/100,000	Diagnostic method
Boberg KM et al.[1]	1998	Norway (Oslo)	25	1995	16.9	1986–1995	1.9	Original criteria[16]
Lee YM et al.[2]	2001	Singapore	24 (11 definite/13 probable)	1990–1996	4	NA	NA	Original criteria
Hurlburt KJ et al.[3]	2002	Alaska natives	49 (42 definite/7 probable)	1983–2000	42.9	NA	NA	Revised criteria
Koay LB et al.[12]	2006	Taiwan	48 (29 definite/19 probable)	NA	NA	2000–2004	0.52	Revised criteria
Wally S et al.[13]	2007	England (West Suffolk)	6	NA	NA	2003–2004	3	Clinical diagnosis
Werner M et al.[4]	2008	Sweden	110	2003	10.7	1990–2003	0.85	Clinical diagnosis
Primo J et al.[14]	2009	Spain (Valencia)	19	NA	NA	2003	1.07	Revised criteria
Jalihal A et al.[5]	2009	Brunei	19	2008	5.6	NA	NA	Revised criteria
Ngu JH et al.[6]	2010	New Zealand (Canterbury)	138 (123 definite/ 15 probable)	2008	24.5 (Age-standardized 18.9)	2008	2.0 (Age-standardized1.7)	Revised/simplified criteria
Haider AS et al.[7]	2010	Australia (Canberra)	42	Unknown	8	NA	NA	Revised criteria
Delgado JS et al.[8]	2013	Israel	100 (73 definite/27 probable)	1995–2010	11.0	1995–2010	0.67	Simplified criteria
van Gerven et al.[9]	2014	Netherland (Amsterdam)	146	1967–2011	18.3	2000–2010	1.1	Revised/simplified criteria
Gronbaek L et al.[10]	2014	Denmark	1,721	2012	23.9	1994–2012	1.68	Clinical diagnosis
Yoshizawa K et al.[11]	2016	Japan (Ueda)	48	2014	23.4	2004–2014	2.23	Revised criteria
Kim et al	2017	Korea	4,085	2009–2013	4.8	2011–2013	1.1	Clinical diagnosis

The wide variation seems attributable to differences between study populations as well as in the identification and definition of cases. In this population-based epidemiological study of AIH in South Korea, cases were identified by ICD-10 code and RID registry information, which are nationwide, comprehensive data sets. The government-run RID registry program is a system for copayment where if a physician diagnoses a patient with AIH based on NHI-provided criteria and enters the ICD code-10 of K75.4, he or she can register the patient for the RID program, which would then provide copayment reduction for the patient. Therefore, we believe the identification of our patients with AIH is reliable.

In other Asian countries, such as Japan and Hong Kong, the frequency of AIH is less than 5% of all chronic active hepatitis cases, whereas in Western Europe and North America, AIH comprises about 20% of these cases.[17] In South Korea, autoimmune liver diseases including AIH have been reported to account for 1% of chronic liver diseases.[18]

The presence of complications or comorbidities was primarily investigated through ICD-10 codes. However, we also examined whether a patient got therapeutic procedures or any relevant medications as ICD-10 codes for complications such as ascites, variceal bleeding or hepatic encephalopathy are often not entered into the claims data system. For example, according to the ICD-10 codes, ascites was present in 3.9% of patients with AIH. However, spironolactone, a drug of choice in the initial treatment of ascites, was prescribed in 11.6%. This range of prevalence of decompensated cirrhosis reflects the limitation of claims data. Hepatocellular carcinoma was diagnosed in 0.8% (31/4,085) of patients. Previously reported frequencies of hepatocellular carcinoma in patients with AIH are 1.1% (8/730 patients),[9] 1.9% (6/322 patients),[19] 3.6% (7/193 patients),[20] 4.0% (10/248 patients)[21] and 6.2% (15/243 patients).[22] Hepatocellular carcinoma frequency mainly depends on the presence of cirrhosis and the duration of the disease.[23] Liver transplantation was performed in 1.1% of our patients. As we could only identify cases of AIH diagnosed with hepatocellular carcinoma or followed by liver transplantation within the 5 year study period, these values may be underreported due to the relatively short follow up period.

A previous nationwide Korean study reported autoimmune thyroiditis as the most common concurrent autoimmune disease, followed by systemic lupus erythematosus.[24] In this study, we could not confirm the diagnosis of autoimmune thyroiditis; however, thyroid hormone was prescribed in 3.6% of patients with AIH. Therefore, it is conceivable that autoimmune thyroiditis was present in 3.6% or less. Out of the 4,085 patients, systemic lupus erythematosus, rheumatoid arthritis, and systemic sclerosis was present in 1.6%, 0.2% and 0.2% of patients, respectively. These were lower compared to previous reports.[25] Corticosteroid or azathioprine was given to approximately 40% of patients, respectively. It seemed to be low; however, analysis was performed for prevalent cases. Prevalent AIH cases in year 2013 possibly include patients who were on remission after treatment for AIH. Previous study also reported that 25% of newly diagnosed patients were not treated.[24]

The strength of the present study is its population-based design. The NHI claims data covers > 95% of the entire Korean population. However, we had some limitations. First, this study was based on insurance claims data. The Korean NHI claims data includes all records of surgery, procedures, tests and medications; however, detailed clinical information such as exact results of tests could not be obtained for each individual patient. Therefore, we were not able to confirm which diagnostic criteria were used, nor could we evaluate clinical features and treatment responses. Some disease codes not responsible for the medical billing could also have been underreported. To complement this limitation, we investigated the prescription rate of concurrent medication or related procedures for complications or comorbidities. Second, the incidence and prevalence of AIH may be underestimated as the actual registration rate of AIH for RID program is not known yet. However, the disease code for AIH (K75.4) is the primary code responsible for the payment and financial benefit and most healthcare institutions and doctors are well aware of the RID program and try to register patients in the program as it offers financial benefits for patients. Third, medical costs may be underestimated because records of some uninsured health services could not be collected. Lastly, the RID registry system has been established since 2009 and this study collected information during a 5 year period, which is limited when evaluating long-term prognoses such as mortality and HCC risk.

In conclusion, this is the first nationwide study on the incidence, prevalence, complications, comorbidities and medical cost of AIH in South Korea. In Korea, the incidence and prevalence were low compared to those of other Western countries with considerable disease burden.

## Abbreviations

AIH: autoimmune hepatitis
ICD: International Classification of Disease
NHI: National Health Insurance
PBC: primary biliary cirrhosis
RID: rare intractable disease
